# Supplementary material for: The distribution and type B trichothecene chemotype of Fusarium species associated with head blight of wheat in South Africa during 2008 and 2009
Source: PLoS One. 2022 Sep 26;17(9):e0275084. doi: 10.1371/journal.pone.0275084 (PMC9512189; doi:10.1371/journal.pone.0275084)
Supplement: Attachment — Submitted filename: Response to Reviewers.pdf [file pone.0275084.s002.pdf]

Response to reviewers

Journal Requirements:

Comment: Please ensure that your manuscript meets PLOS ONE's style requirements, including those for file naming.
Response: This has been addressed

Comment: In your Methods section, please provide additional location information about your study sites, including geographic coordinates for the data set if available
Response: GPS coordinates and elevation (m) for each location has been added in Table 1

Comment: Please review your reference list to ensure that it is complete and correct. If you have cited papers that have been retracted, please include the rationale for doing so in the manuscript text, or remove these references and replace them with relevant current references. Any changes to the reference list should be mentioned in the rebuttal letter that accompanies your revised manuscript. If you need to cite a retracted article, indicate the article's retracted status in the References list and also include a citation and full reference for the retraction notice.
Response: The reference list is complete and correct. No cited papers have been retracted. The following changes was made to the reference list:

1. Due to a comment by the second reviewer ("The introduction and discussion are a bit long. I suggest to reduce some content not well fit the key points."), the section containing reference no 36 in the Introduction was deleted, along with reference no 36. Also, the section containing references no 80 (Von der Ohe *et al*., 2010), 81 (Puri and Zhong, 2010) and 83 (Albuquerque *et al*., 2021) in the Discussion was deleted, along with these references.
2. Reference no 48 (a reference to a website) was replaced with a more suitable reference, namely a scientific publication (Zhao *et al*., 2018).
3. Reference no 64 (O'Donnell *et al*. 1998) was erroneously cited as reference no 64, while it should have been reference no 55. This was rectified.
4. Reference no 57 (Ward *et al*., 2008) was erroneously cited as reference no 57, while it should have been reference no 63. This was rectified.
5. One reference was added to the reference list, namely reference no 72 (Drakopoulos *et al*., 2020).
6. The order in which references was cited in the manuscript was updated according to the changes mentioned, and is shown with track changes in the Revised Manuscript with Track Changes.
7. DOI's have been added to references.

Additional Editor Comments

Comment: Please (if necessary) change the significant numbers in table 3, when less than 100 samples are taken, you cannot have a % incidence of lower than 1%...so something like 36.36 is impossible, should be then 36.
Response: I hope that I understand the comment correctly. In total 80 symptomatic wheat heads were sampled at each locality, unless indicated otherwise in the Materials and Methods. Thus, if for example, the total number of *Fusarium* isolates obtained at a locality

was 30 FGSC isolates and five FIESC isolates, then a total of 35 *Fusarium* isolates was obtained in the 80 wheat heads at that locality. The incidence of the *Fusarium* spp. was then calculated as follows: For FGSC: (30/35) x 100 = 85.71%, and for FIESC: (5/35) x 100 = 14.29%. This is also indicated in the subscript of Table 3.

Comment: The chemotypes presented in a table is not clear. It would be better to somehow also include a pie diagram put on the geography map. It is known from previous work by Waalwijk and coworkers that geography (e.g. altitude) can affect the chemotype.

Response: Pie charts indicating the incidence of type B trichothecene chemotypes of different members of the *Fusarium sambucinum* species complex (FSAMSC) obtained, are presented on a separate geography map (Fig. 2). The original Table presenting the chemotypes (Table 4) has been deleted.

Comment: There are quite some differences between the western cape, free state, and the rest...the reason for these differences are now somehow addressed in the discussion, but in my opinion would merit a section in the results.

Response: This was addressed under "Geographical distribution of *Fusarium* species", in lines 335 – 404 of the Results ("All mark-up" under "Track Changes" in the Revised Manuscript with Track Changes), where species diversity and incidence over years at regional level was discussed, as well as species diversity and incidence at individual localities over years. If anything more specific is required, I am happy to address it.

Comment: Did you look at co-occurence? recent insights (study by tan et al., 2021 and some reports before by several authors) report on the tendency of some species to co-occur in disease complexes. Did you observe any of these co-occurences as they might be one of the important drivers explaining how the plasticity of a population works.

Response: Co-occurrence of *Fusarium* species was very low, occurring in only 0.9% of isolations performed. This is mentioned in the Results in lines 330 – 333, and in the Discussion in lines 517 – 519 (both with "All mark-up" under "Track Changes" in the Revised Manuscript with Track Changes).

Comment: What about climatic conditions? can your report be put in a historic context? have you seen changes compared to previous report?

Response: None of the previous studies done in South Africa included any climatic conditions, it is therefore unfortunately not possible to compare our study with previous studies.

Review Comments to the Author (second reviewer)

Comment: The high frequency of FIESC in this study seems strange for me. Normally it is a very weak pathogenic species to wheat and widely distributed in soil, plant debris, surface of rice and wheat grains and so on. In my experience, for the wheat head samples with obvious symptoms, the isolation frequency would be lower than 1%, which can be ignored in the field FHB management practice. To my knowledge, there is also no report of such high ratio of FIESC as FHB pathogen before in other place worldwide. So the author should be careful about if they are real the causal agent of FHB or just surface contamination on the heads. Details of the isolation procedure should be examined. For example, for the heads with very mild symptom (i.e. without red mold), it would be hard to find the infected spikelet when they were dried several days after sampling, this maybe

influence the result of the isolation. Anyway, I suggest the authors discuss the reasons of the unusual observation, if they are the pathogen, the high frequency of FIESC should considered in the field control and mycotoxin contamination.

Response: Reasons for the high occurrence of the FIESC are provided in the Discussion, from line 501 onwards (“All mark-up” under “Track Changes” in the Revised Manuscript with Track Changes). A subsequent study was conducted by our group on the FIESC isolates obtained in this study, to determine species diversity and toxigenic potential, and is due for publication. Since this study revealed isolates of the FIESC obtained in this study to have low toxigenic potential, it was decided to delete the following sentence from lines 513 – 517 (“All mark-up” under “Track Changes” in the Revised Manuscript with Track Changes): “Its abundance in South Africa is a cause for concern, as the FIESC has been associated with a range of mycotoxins such as the trichothecenes DAS, NIV, T-2 toxin, as well as beauvericin, butenolide and ZEA [10, 55]. It is, therefore, imperative that the identity of members of the FIESC, their respective toxigenic potentials, as well as their impact on the South African grain industry be resolved.” It was subsequently replaced with the following sentence in lines 522 – 525 (“All mark-up” under “Track Changes” in the Revised Manuscript with Track Changes): “A subsequent study on the FIESC isolates obtained in this study revealed high species diversity, but low toxigenic potential (unpublished data), indicating that this species complex may be less important as FHB pathogens in South Africa.”

Comment: P14L283-285, FCSC1, FCSC5 and FSSC5 were found. Did they determine by EF1a blast? These species were identified by phylogenetic analysis of several conserved genes, only ef1a sequence is identical?

Response: Yes, those were identified via sequencing of TEF1 followed by BLAST analyses as described in the Materials and Methods. Species circumscription based on Genealogical Concordance Phylogenetic Species Recognition (GCPSR) requires sequencing and analyses of multiple loci, but TEF1 has proven effective for identification of many *Fusarium* species and is widely used for this purpose.

Comment: The introduction and discussion are a bit long. I suggest to reduce some content not well fit the key points.

Response: Content that does not fit well in the Introduction and Discussion have been deleted. Please see track changes in the Revised Manuscript with Track Changes

Comment: I noticed that the sample size of the places where *F. pseudograminearum* was predominant are small. The authors should mention it in the discussion, because small sample size is easy to cause bias.

Response: A subsequent study found that *F. pseudograminearum* was the dominant species obtained from symptomatic wheat heads at three localities in the Western Cape, constituting more than 80% of ~300 isolates. This is mentioned in lines 529 – 532 of the Discussion (“All mark-up” under “Track Changes” in the Revised Manuscript with Track Changes). These results are not included in this manuscript, since it forms part of a separate study into population genetics of *F. pseudograminearum*, due for publication.

Comment: Adding line numbers after page 21 will facilitate review.

Response: Line numbers have been added from the beginning to the end of the manuscript.
