# Supplementary material for: The distribution and type B trichothecene chemotype of Fusarium species associated with head blight of wheat in South Africa during 2008 and 2009
Source: PLoS One. 2022 Sep 26;17(9):e0275084. doi: 10.1371/journal.pone.0275084 (PMC9512189; doi:10.1371/journal.pone.0275084)
Supplement: Attachment — Submitted filename: Response to Reviewers.docx [file pone.0275084.s003.docx]

**Response to reviewers**

*Journal Requirements:*

Comment: Please review your reference list to ensure that it is complete and correct. If you have cited papers that have been retracted, please include the rationale for doing so in the manuscript text, or remove these references and replace them with relevant current references. Any changes to the reference list should be mentioned in the rebuttal letter that accompanies your revised manuscript. If you need to cite a retracted article, indicate the article’s retracted status in the References list and also include a citation and full reference for the retraction notice.

Response: The reference list is complete and correct. All reference were checked on the PubMed database or the journal’s website to confirm that it has not been retracted. To the best of our knowledge, none of the references cited has been retracted. The personal communication with Richard Findlay (p. 29, line 535) has been replaced with two scientific articles (references 81 and 82), and the reference list has been updated accordingly. All Journal names have been edited to their correct abbreviations according to NCBI database, and all URLs and doi’s have been removed.

*Additional Editor Comments*

Comment*:* There is still one minor comment, in regard to table 3: the calculated incidence: "then calculated as follows: For FGSC: (30/35) x 100 = 85.71%, and for FIESC: (5/35) x
100 = 14.29%. This is also indicated in the subscript of Table 3." But my point is that when you have 100 sampels, you can never been more precise than 1%, so for example 1.567% is impossoble. This also accounts when you work with less samples, so everything lower than 1 shoudl definetely be removed....85.71% should be 85%...this is as precise as you can be when doing these types of calculation.

Response: This has been addressed accordingly.

*Reviewer’s Comments*

Comment: While revising your submission, please upload your figure files to the Preflight Analysis and Conversion Engine (PACE) digital diagnostic tool, <https://pacev2.apexcovantage.com/>. PACE helps ensure that figures meet PLOS requirements. To use PACE, you must first register as a user. Registration is free. Then, login and navigate to the UPLOAD tab, where you will find detailed instructions on how to use the tool. If you encounter any issues or have any questions when using PACE, please email PLOS at [figures@plos.org](mailto:figures@plos.org). Please note that Supporting Information files do not need this step.

Response: Figure files (Fig. 1 and Fig. 2) has been uploaded to the PACE digital diagnostic tool and has been archived.
