# Supplementary material for: The distribution and type B trichothecene chemotype of Fusarium species associated with head blight of wheat in South Africa during 2008 and 2009
Source: PLoS One. 2022 Sep 26;17(9):e0275084. doi: 10.1371/journal.pone.0275084 (PMC9512189; doi:10.1371/journal.pone.0275084)
Supplement: Attachment — Submitted filename: Response to Reviewers.docx [file pone.0275084.s005.docx]

**Response to reviewers**

*Journal Requirements:*

Comment: Please review your reference list to ensure that it is complete and correct. If you have cited papers that have been retracted, please include the rationale for doing so in the manuscript text, or remove these references and replace them with relevant current references. Any changes to the reference list should be mentioned in the rebuttal letter that accompanies your revised manuscript. If you need to cite a retracted article, indicate the article’s retracted status in the References list and also include a citation and full reference for the retraction notice.

Response: The reference list is complete and correct. All references were checked on the PubMed database or the journal’s website to confirm that it has not been retracted. To the best of our knowledge, none of the references cited has been retracted.

*Reviewer’s comments:*

*6. Review Comments to the Author*

**Reviewer #2:**

- Title and Abstract: a little bit misleading and unclear title. Since this survey was done in 14 years ago, it is better point out the time period in the title and abstract. Otherwise, audience will think this is a latest survey report.

Response: The title has been changed to reflect the time period in which the survey was conducted. This time period is also indicated in the Abstract. The Abstract has been reduced to 300 words, in accordance to Journal requirements.

- Discussion: since this survey study is from 2008/09, almost 15 years ago. it is better to see if there is a latest survey report published recently and make a comparison.

Response: To the best of our knowledge, from the time this survey was conducted, no newer surveys were done or reported in South Africa. Our survey is, therefore, the latest and most complete report on *Fusarium* species associated with FHB of wheat in South Africa to date. All previous surveys done in South Africa, and results obtained, are mentioned in the Introduction and thoroughly discussed in relation to our findings in the Discussion. In the most recently published survey (Minnaar-Ontong *et al*., 2017), no mention is made of when their study was conducted, while the isolates they obtained was identified using morphological characteristics only. Minnaar-Ontong *et al*. (2017) also obtained samples from fewer locations and production regions than our study. Every study to date conducted under South African conditions is comprehensively referred to in the Discussion.

- Line 443: this is not a "current" study, but almost 15 years old

Response: This has been addressed (line 449-450 in the Revised Manuscript with Track Changes – “Simple Markup” in the “Review” Tab).

**Reviewer #3:**

- Your manuscript titled "The distribution and type B trichothecene chemotype of Fusarium species associated with head blight of wheat in South Africa" is a well written and structured manuscript that provides valuable information on the species associated with FHB infection. Unfortunately, the research and findings are outdated. Utilizing words such as "first report" is not accurate given that the sampling has been completed 13 and 14 years ago. The distribution pattern of species changes over the time while facing the changes of cropping system and practices, environment, the level of resistance of cultivars grown etc. I believe many changes could have happened in the last 14 years that could have changed the distribution pattern you are reporting in this study. Your manuscript still has a great value because it reports what species were predominant over a decade ago which could be a reference for newer studies. I strongly encourage you to address the findings as an overview research over the late 2000s.

Response: Just prior to first submitting this manuscript to PLOS ONE on 2021/09/03, the generated *EF1-α* sequences were compared to existing sequences in the databases mentioned in the Materials and Methods, in collaboration with our co-author, Dr. Todd Ward (Director – ARS Culture Collection (NRRL)) from the USDA. Results were then compared to existing literature, to ensure that the first reports of *Fusarium* species mentioned in the manuscript are valid. That is how, for example, the first report of *Fusarium transvaalense* from wheat was provided, even though this species was only described in 2018 (reference # 70: Sandoval-Denis *et al*., 2018). This is also how the potentially novel *Fusarium* spp. from the FCSC, FFSC, FOSC, FSAMSC and FTSC was identified.

The *Fusarium* species associated with FHB in South Africa could have changed in response to changes of cropping system and practices, environment, the level of resistance of cultivars grown etc. Therefore, and due to the fact that our study was conducted from 2008 – 2009, care is taken to present our study as originating from the late 2000s, while emphasising the need for new studies, since our study is still the latest and most complete survey done in South Africa to date. (see Abstract, Introduction (lines 137-140), Materials and Methods (lines 160-162 and 176-180) and Discussion (lines 438-439, 473-475, 480-490, 494-500, 501-503, 574-589, 590-598) in the Revised Manuscript with Track Changes – “Simple Markup” in the “Review” Tab).

**Reviewer #4:**

This paper reports survey results conducted to determine the identity, distribution and type B trichothecene chemotype of Fusarium species associated with FHB of wheat in South Africa.
• General concern: The survey was in 2008 and 2009 which is about 13-14 year ago making it a historical data. So it doesn’t show recent developments although it still provides a good information if documented as a publication.

Response: No response needed.

Abstract:

- The finding shows chemotype diversity was limited (15-ADON=90.1%) and highly structure in terms of species difference. However, authors report for the first time six Fusarium species not recorded on wheat elsewhere which is a plus.

Response: No response needed.

- Results were not indicated as related to production conditions – irrigated versus non-irrigated and province wise. One thing that should be addressed here is whether the species distribution under different farming conditions and provinces the same or different? Please describe it briefly.

Response: This has been addressed in the Abstract (lines 33-35 in the Revised Manuscript with Track Changes – “Simple Markup” in the “Review” Tab). The Abstract has also been reduced to 300 words, in accordance to Journal requirements.

Materials and Methods:

- There is no mention if the locations overlapping across the two survey years. Please describe this as *F*. species differences between the years could be a factor of the sites of sample collection besides crop culture.

Response: This has been addressed (lines 162-165 in the Revised Manuscript with Track Changes – “Simple Markup” in the “Review” Tab).

Results:

- Line#273: What are the factors behind such large differences in the prevalence of FGSC between 2008 and 2009? This should be discussed in terms of sites of sample collection, weather condition, crop culture / rotation crops or species competition.

Response: This has been addressed in the Discussion (lines 574-587 in the Revised Manuscript with Track Changes – “Simple Markup” in the “Review” Tab):

“FGSC and FIESC isolates were more abundant in the Free State in 2008 compared to 2009. This also happened for the FGSC in the Northern Cape. In KZN, the occurrence of the FGSC remained almost unchanged during the two years. The presence of all members of FCSC, *F*. *temperatum* and *F*. *verticillioides*, *F*. *oxysporum* (FOSC), *F*. *armeniacum*, *F*. *culmorum*, *F*. *lunulosporum*, *F*. *transvaalense*, FSSC 5, *F*. *acuminatum*, *F. avenaceum*, and an unknown *Fusarium* sp. within FTSC in 2008 but not 2009, and *F*. *subglutinans*, *F*. *brachygibbosum*, and unknown *Fusarium* spp. within FFSC and FSAMSC in 2009 but not 2008, may have been coincidental since the incidence of all of these species was very low. The high incidence of *Alternaria* species obtained in all production regions in 2009 might have contributed to lower *Fusarium* levels in the 2009 production season, while differences in climate, cropping history and agronomic practices of the collection sites in the two years might have also contributed to the discrepancy in *Fusarium* species composition between years [40, 41, 43]. Variation in the timing and the amount of water provided through irrigation, especially near anthesis, could also have influenced the resultant disease intensity and associated *Fusarium* species between years [87].”

- Fig. 1. FGSC is common across all regions with one exception. In Western Cape and followed by Free State the proportion of *F*. *pseudograminearum* is exceptionally high. What makes this region so special. This needs discussing (seemingly due to bias in small sample size).

Response: This has been addressed in the Discussion (lines 501-512 in the Revised Manuscript with Track Changes – “Simple Markup” in the “Review” Tab):

“An interesting observation was the dominance of *F*. *pseudograminearum* as an FHB pathogen at one locality in the eastern Free State and the one locality in the Western Cape. The dominance of this species at Bethlehem in the eastern Free State may be due to it being introduced earlier via infected seed [35, 85], especially since *F*. *pseudograminearum* was not found at any other locality in this region. Although the sample size in the Western Cape was quite small (one locality with 40 wheat heads sampled), a study conducted more recently from three localities in the Western Cape revealed that *F*. *pseudograminearum* was the dominant species obtained from wheat heads exhibiting FHB symptoms at all three localities, constituting more than 80% of ~300 isolates obtained (unpublished data). *Fusarium pseudograminearum* is best known as the cause of Fusarium crown rot (FCR) of wheat [73, 74]. Its dominance in the Western Cape can be ascribed to the prevalence of FCR in this region as well as the use of minimum / no till practices, which results in a build-up of inoculum levels in stubble [74].”

The more recent study, in which ~300 *F*. *pseudograminearum* isolates was found from FHB at three localities in the Western Cape (unpublished data), forms part of a population genetics study currently being conducted within our research group, which will be published in future.

- Lines#443-444: This sentence is confusing. Were these six Fusarium species not recorded on other crop species similar to wheat? If they reported on other plant species, they are not new species. They could be a new record on a wheat crop. Please reword the sentence.

Response: This has been addressed (line 449-450 in the Revised Manuscript with Track Changes – “Simple Markup” in the “Review” Tab).

- Please check references cited.

Response: The references cited in the text and reference list are complete.
